# Lecture on Chloral Hydrate

**Published:** 1872-09

**Authors:** J. H. Etheridge

**Affiliations:** Prof. Materia Medica and Therapeutics, in the Rush Medical College; Chicago


Article IV.—*Lecture on Chloral Hydrate.* By J. H. Etheridge, M.D., Prof. Materia Medica and Therapeutics, in the Rush Medical College.

Gentlemen—I wish this afternoon to call your attention to an unofficinal preparation which is just now claiming much attention. It is *Chloral Hydrate*, and the evidences daily accumulating in its favor from all quarters of the globe, indicate that the expensive, and, thus far, indispensable opium, is soon to find a powerful rival that may completely supplant it.

Nearly forty years ago Liebig discovered this preparation, and until within three or four years it has remained a chemical curiosity. To Dr. Liebreich, of Berlin, are we indebted for a knowledge of its therapeutic value.

Chemically, chloral is tri-chlorinated aldehyd, *i. e.* aldehyd in which three of the hydrogen atoms have been replaced by an equal number of atoms of chlorine, and its composition is represented by the formula $C_2HCl_3O$. It is prepared by passing a stream of chlorine gas through absolute alcohol, until the entire contents of the flask is converted into a solid, white, crystalline mass. For this purpose the chlorine gas must be forced through the alcohol 60 or 70 hours. The contents of the flask is then purified by sublimation, which yields a snow-white, dry, crystalline powder. Treated with an alkali, chloral and its hydrate yield chloroform and a formate, ($C_2HCl_3O+NaHo=CHCl_3+NaCHO_2$). Upon this fact is based the theory that this drug is converted into chloroform in the circulation, and thus is produced its peculiar effect. From this reaction of an alkali we also obtain a safe and reliable test for the purity of chloral. To a solution of potassa add some chloral; if the latter be pure, it *will not color the solution, and the smell of pure chloroform will be emitted.* If a brown precipitate or color be formed, or if pungent gases be evolved, the chloral is impure and dangerous.

Therapeutically, chloral hydrate is a nervous sedative.

Dr. Strothers states that locally it is of excellent service in "neuralgia, pleurodynia, rheumatism, gastralgia, nausea and vomiting." A saturated aqueous solution is applied over the painful part, with slight friction and glycerine; olive oil or cream is used as a subsequent dressing. Thus used some of it is probably absorbed, and a rubefacient action is also produced.

Hypodermically, it is *not* to be used, because of its great liability to produce abscesses.

It is inadmissible in organic heart and brain disorders.

Its effect on the alimentary canal is not prominently noticeable. It neither vomits nor purges as a rule.

Its effect on the heart is at first to increase its action; subsequently the heart beats are normal in frequency. The animal heat is decreased, and thereby it is inferred that tissue metamorphosis is arrested. Long continued use of it is said to injure the health quite seriously "by making the blood watery." Prof. N. R. Smith, of Baltimore, mentions some cases where chloral had been taken for a long time, daily, followed by desquamation of cuticle, and acute ulcerations of the fingers, particularly about the nails, attended by hyperæsthesia, accelerated pulse, and general malaise. The Professor regards the long-continued administration of this drug as productive of toxical effects somewhat similar to those produced by ergot.

Perspiration is diminished, and the effect on the genito-urinary apparatus is at first to decrease the solid constituents of the urine, and increase relatively the watery portion. Subsequently the elimination of solids is considerably increased for several hours.

Its chief effect is manifest on the cerebro-spinal centres. Hammond claims that its first effect is to increase the amount of blood in the brain, and this is quickly followed by cerebral anemia, which continues till the patient awakens. You readily recall to mind the detailed description I gave you early in the course, of his (Hammond's) mode of procedure in investigating the effect of the bromides on cerebral circulation. The patient experiences relief from small pains, as of neuralgia, in lighter forms, in eight or twelve minutes, and if the dose be large enough, sleep ensues in about twenty minutes. In very severe cases of pain, or delirium tremens, sleep cannot be procured sometimes for several hours. The range varies from a few moments to hours, before hypnotism is secured.

Common nervous headache is frequently relieved by 10-gr. doses. Be sure your case is one of what may be called "nervous" headache, before giving chloral.

Neuralgia, generally, in its various forms, has been relieved by 20 grs. or more, repeated once in two or three hours. I expect, however, to tell you a better method of controlling this affection in its severe forms, when we come to speak of hypodermic injections.

Neuralgic rheumatism, gout, and inflammatory rheumatism, are stripped of their terrors by administration of this drug.

Choreic manifestations are most excellently controllable by chloral; and its effects are particularly prominent in those cases where the patient is in danger of dying from exhaustion produced by the constant and violent jactitation.

After *all injuries* accompanied by pain, this agent can be profitably used. During the late Franco-German war, English surgeons used chloral in nearly every case. These surgeons left their homes in England of purpose to follow in the rear of each army, and after battles they assisted in caring for the wounded. In many instances the wounded, being very numerous, would lie nicely for hours under the influence of chloral, free from pain, awaiting their turn for the surgeon to amputate or dress, as their cases demanded.

The atrocious pains of nephritic or biliary colic can be effectually quieted by this drug.

As a palliative in malignant diseases, it is extensively used; 10 grs. thrice daily, answers in the worst cases. In many cases, double that dose at bedtime, only suffices.

Superintendents of insane asylums regard it as the king of soporifics, in procuring sleep and quietness, and consequent amelioration in some forms of insanity.

Closely allied to this affection is delirium tremens, and evidence is daily accumulating of the superiority of chloral over every other remedy in securing sleep. In this condition the doses must be large, and it is astonishing to see what immense doses can be borne. In recent cases the dose need not be over one scruple every two hours, but where the delirium and sleeplessness have continued many days and nights, the difficulty is much greater in inducing sleep, and the danger of the patient at best is imminent, for experience teaches that such subjects are liable to die with or without medication; hence you will find such patients in some cases bearing drachm doses hourly, till sleep supervenes, and subsequently awakening sane and weaker. You will, occasionally, meet with cases where death will result with the lightest treatment. Extreme cases of this affection need prompt, vigorous treatment from the very first, or they will die in spite of every effort an intelligent physician can put forth.

In obstetric practice, many physicians have used chloral with the happiest effect. Given in cases of rigidity of the os during labor, with irregular ineffectual pains, in sufficient quantity to produce sleep and check pains, it is followed by recuperation of strength, and patient is awakened by severe pains which result in speedy delivery. Dr. Da Cunha, of Bombay, gives three cases of retarded labor in which he used half-drachm doses to procure sleep, which were followed by sharp pains after a refreshing sleep, and a speedy delivery in each case. It has also been given in the first stage very frequently, where there was much restlessness and exhaustion present. The evidence of its good service is daily increasing in puerperal convulsions. Sometimes all remedies fail but chloral—in large doses—in puerperal mania.

Given in five-grain doses, hourly, it will usually relieve hiccough of prolonged and dangerous character.

In asthma its success is doubtful.

Cough of measles (from bronchitis), which is especially troublesome in children, is frequently quickly checked.

In tetanus of new born children, it has been used with good results, given in one or two-grain doses, on the occurrence of each convulsion. It is also recommended in grain doses in infantile colic, but my experience in its use in this troublesome affection does not lead me to recommend it very strongly.

In traumatic tetanus it is almost absolutely sure of answering its desired end. Herein it succeeds when other remedies fail, and it must be given heroically.

I have now enumerated only a portion of the diseases in which chloral is used. Many experiments in various directions have been tried—as in minor surgical operations (one case of amputation of the leg is reported, even), in the exanthematous fevers, in incontinence of urine, in spermatorrhœa, etc., etc., but the diseases I have enumerated seem to be the principal ones to which its beneficient effects are now chiefly confined.

One point I must here allude to before proceeding farther, and that is, the alarmingly increasing indulgence in this substance as a narcotic luxury. Men and women who suffer from sleeplessness habitually, are easily tempted to resort to it, and many, very many do it. Amongst its votaries are business men harassed with great anxieties, school-teachers, book-keepers, invalid women made weaker by family cares; in each of these instances I have seen chloral victims. This bad habit is not yet so prevalent as opium-eating, or as habitual bromide-taking of which I recently spoke to you.

### VEHICLE AND POSOLOGY.

The menstruum should be large, as the effect on the mouth and fauces is almost escharotic. Some aromatic syrup is the pleasantest vehicle to take it in. My usual prescription is two scruples to the ounce, and then a teaspoonful or dessertspoonful gives five or ten grains as desired.

The dose must be graduated to the case; drachm doses to a baby, or grain doses to an inebriate are manifestly incongruous. Physicians have, until recently, generally said, "There *is no dose* of chloral." Some will tell you, "I get uniformly good results from fifteen-gr. doses," and others, "I never use less than forty-five grains in severe cases to start with, and subsequently, as needed, and I have never yet found any ill results following." In my own practice, however, I have settled down to scruple doses, excepting in delirium tremens and tetanus. In these two cases I give much larger doses. The severity of the cases must be your guide. The enormous doses borne by mania a potu and tetanic patients is simply astonishing. The physicians of the Royal Infirmary of Edinburgh, administer it in thirty and sixty-grain doses every half hour, repeated three or four times, "and a deep and lengthened sleep generally ensues, the general result being," they say, "to keep the wards almost empty from the rapidity with which the patients are enabled to be discharged."

Dr. Landsdown of the "Bristol General Hospital," reports two cases of tremens in which the beneficial effects of large doses are shown. To the first case he gave sixty grains, and "this made him drowsy and nothing more;" in thirty minutes, sixty grains more were given, and he slept two hours. The second case—a female—was treated in a similar manner (two one-drachm doses given thirty minutes apart), and in an hour after the second dose, another drachm dose was given per rectum. She then slept heavily and was soon discharged.

In tetanus, the doses are not so large at first, but they are *often repeated*, so that in the aggregate as much or more is taken; usually, thirty or forty grains are given to start with, and then fifteen or twenty grains are given every two hours according to the indications.

A case is recently related in the London Lancet where a man took seven drachms (420 grains) and no bad results followed. This amount was all taken in one night. Another case is reported by Dr. P. C. Williams, of Baltimore, where the "patient took about 600 grains (10 drachms), the only appreciable effect of which was to produce coma, lasting 18 hours, and ending in complete recovery without any medicinal treatment whatever, during the state of coma."

I can hardly believe that these enormous doses are all absorbed by the alimentary canal within an hour or so. Possibly they were absorbed slowly, thus prolonging the sleep, and were rapidly eliminated by the lungs and skin.

Dr. Richardson, of London, regards 120 grains (2 drachms) as the maximum dose, "dangerous but not of necessity fatal. Beyond one hundred and twenty grains," he continues, "the danger increases, and one hundred and eighty grains may be considered a dose that would in the majority of cases prove fatal." I quote this author because he is doubtless the best living authority we have on the effects on the human system of the organic compounds.

Given with an alkali preparation (in syrup) chloral is said to act much more rapidly—probably because the alkali facilitates decomposition of the chloral and the absorbing process.

Much more might be said of this wonderful drug, but our hour is drawing to a close. At our next meeting, I shall compare this drug with opium, and indicate some of its superiorities.

